# Role of plaque imaging for identification of vulnerable patients beyond the stage of myocardial ischemia

**DOI:** 10.3389/fcvm.2023.1095806

**Published:** 2023-03-17

**Authors:** Ryoko Kitada, Kenichiro Otsuka, Daiju Fukuda

**Affiliations:** Department of Cardiovascular Medicine, Osaka Metropolitan University Graduate School, Osaka, Japan

**Keywords:** chronic coronary syndrome, acute coronary syndrome, imaging, ischemia, atherosclerosis, plaque vulnerability, coronary microvascular dysfunction, inflammation

## Abstract

Chronic coronary syndrome (CCS) is a progressive disease, which often first manifests as acute coronary syndrome (ACS). Imaging modalities are clinically useful in making decisions about the management of patients with CCS. Accumulating evidence has demonstrated that myocardial ischemia is a surrogate marker for CCS management; however, its ability to predict cardiovascular death or nonfatal myocardial infarction is limited. Herein, we present a review that highlights the latest knowledge available on coronary syndromes and discuss the role and limitations of imaging modalities in the diagnosis and management of patients with coronary artery disease. This review covers the essential aspects of the role of imaging in assessing myocardial ischemia and coronary plaque burden and composition. Furthermore, recent clinical trials on lipid-lowering and anti-inflammatory therapies have been discussed. Additionally, it provides a comprehensive overview of intracoronary and noninvasive cardiovascular imaging modalities and an understanding of ACS and CCS, with a focus on histopathology and pathophysiology.

## 1. Introduction

Coronary artery disease (CAD) is a progressive disease that often first manifests as acute coronary syndrome (ACS). ACS is a life-threatening disease that affects approximately 1,045,000 people per year in the United States, leading to hospitalization and contributing to a remarkable economic and health care burden (1). In 2019, the European Society of Cardiology proposed the term chronic coronary syndrome (CCS) in the revised guidelines for stable CAD (2). It emphasizes that CCS is a chronic condition rather than the conventional “stable” angina or “stable” CAD. This paradigm shift calls for the early diagnosis, intervention, and continuous treatment of risk factors in patients with CCS to prevent cardiovascular events, including sudden death, ACS, and heart failure.

Clinical symptoms are an important aspect in diagnosing CCS (2). Although ACS often occurs as the first manifestation, noninvasive tests such as electrocardiography, echocardiography and elevated cardiac troponin levels can help in the diagnosis of ACS (3). Imaging modalities are highly useful for assessing the presence of CAD in patients with and without clinical symptoms based on clinical risk factors. The therapeutic goals of CCS include the lifelong prevention of ACS development and symptom reduction (2). While the primary percutaneous coronary intervention (PCI) strategy reportedly improves outcomes in patients with ST-segment elevation myocardial infarction (STEMI) (2), the evaluation of myocardial ischemia, a surrogate marker of the disease, is central to determining indications for coronary revascularization in patients with CCS. The COURAGE (the Clinical Outcomes Utilizing Revascularization and Aggressive Drug Evaluation) trial demonstrated that PCI did not reduce the risk of death, nonfatal myocardial infarction (MI), or other major cardiovascular events (4). Subsequently, a sub-analysis demonstrated that a greater ischemic burden is associated with PCI benefits (5). To determine whether PCI is superior to optimized medical therapy (OMT) in patients with stable angina and a high ischemic burden the International Study of Comparative Health Effectiveness With Medical and Invasive Approaches (ISCHEMIA) trial was conducted at 320 institutions in 37 countries (*n* = 5,179). The ISCHEMIA trial compared the invasive (OMT + coronary revascularization) and conservative (OMT + invasive strategy, if necessary) strategies in patients with stable angina pectoris and moderate-to-severe myocardial ischemia (>10%), while excluding patients with heart failure, left main coronary lesions, and chronic kidney disease (6). There was no statistically significant difference in the primary endpoint between the two groups during follow-up. The observations from these key clinical trials indicate the importance of OMT and coronary revascularization with their appropriate timing and indications.

**Figure 1:**
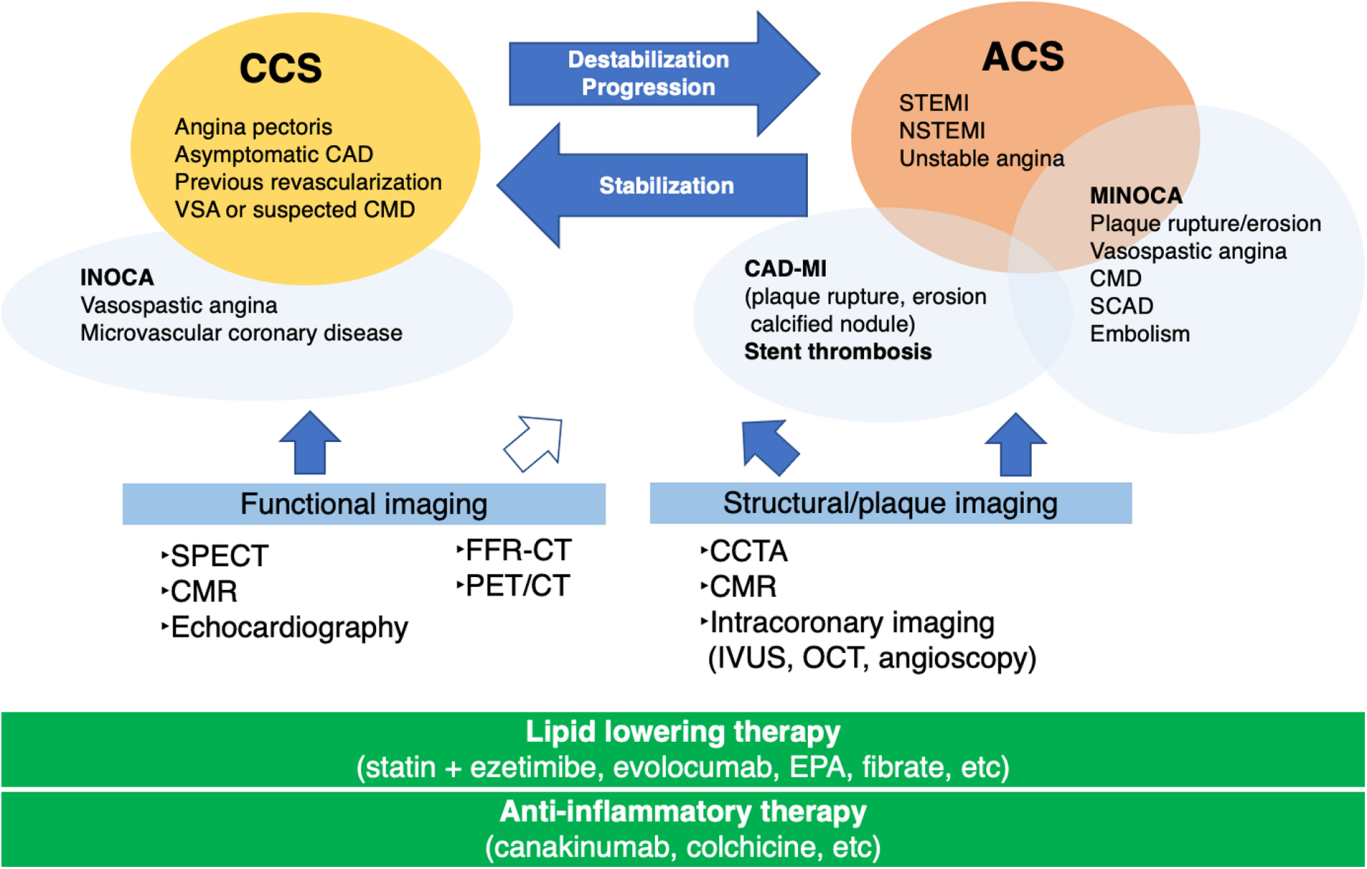
Role of imaging in the management of patients with coronary artery disease.

The identification of underlying factors, such as coronary plaque burden, high-risk plaques, and coronary microvascular disease (CMD), is crucial for the management of patients with CCS beyond the stage of myocardial ischemia. Figure 1 illustrates the role of imaging and therapeutic targets in the management of patients with CAD. Imaging modalities are clinically useful in deciding the appropriate management of patients with CAD (2, 7–11). The development of noninvasive imaging technologies, such as coronary computed tomography angiography (CCTA) and cardiac magnetic resonance (CMR), has propelled our understanding of the features that accelerate subclinical CAD leading to ACS (12, 13). Intracoronary imaging has deepened our understanding of the mechanisms underlying coronary lesion destabilization in ACS (14, 15). Accurate prediction of coronary lesions leading to ACS requires a comprehensive assessment of plaque vulnerability, including plaque burden (16, 17), inflammatory status (18), coronary plaque mechanical stress (19, 20), and coronary microvascular function (21). These in turn determine the fate of coronary plaque rupture/erosion. In this study, we present a review highlighting the latest CAD knowledge and investigate the role and future perspective of coronary plaque imaging in the diagnosis and management of patients with CAD and clinical trials for lipid-lowering and anti-inflammatory therapies.

## 2. Pathophysiology of ACS

Understanding the pathogenesis of ACS largely relies on careful autopsy studies of sudden cardiac death. Fuster et al. proposed the concept of ACS, including unstable angina, acute MI, and sudden cardiac death, and determined that plaque rupture-induced thrombus formation is linked to occlusion and severe stenosis of the coronary arteries (3, 22). The most common pathological cause of ACS is plaque rupture; postmortem studies conducted in the 1980s demonstrated that plaque rupture is found in the most fatal MIs (23, 24), indicating the presence of vulnerable plaques. Vulnerable plaques are typically characterized by a large central lipid core which is covered by a thin inflamed fibrous cap with few smooth muscle cells (25); chronic inflammation weakens the collagen structure of the fibrous caps (19, 26). In recent decades, attempts have been made to use imaging to identify precursor lesions that progress to ACS (16, 27–30) on the hypothesis that local therapeutic interventions may prevent plaque rupture-induced thrombosis. Increased plaque volume (31), thin fibrous caps (32), and microcalcifications (27, 33) detected on imaging modalities reportedly serve as independent predictors of ACS; however, ACS prediction remains challenging (34).

Plaque erosion is the second leading cause of ACS, is reportedly more common in women, and has fewer inflammatory cells and proteoglycan-rich lesions than a ruptured plaque (35–38). Superficial erosion-induced plaque thrombosis involves less macrophage-mediated inflammation, just as in the case of fibrous cap rupture. Superficial erosion is complicated by lesions with different epidemiologies and morphologies and involves a pathophysiological mechanism different from that of a fibrous cap rupture. Factors other than plaques, such as endothelial shear stress and neutrophil extracellular traps (NETosis) (37, 38), are thought to play a vital role in plaque erosion-induced thrombus formation. Furthermore, recent clinical studies have demonstrated that intimal healing following a silent plaque rupture/erosion plays a vital role in plaque progression (39, 40).

Following plaque rupture and erosion, calcified nodules are reportedly the least common cause of ACS (≤5%). Calcified nodules are characterized by the eruption of calcified nodules with an underlying fibrocalcific plaque and minimal or no necrosis (25). ACS caused by plaque erosion or calcified nodules can cause non-occlusive thrombosis in the culprit lesion; however, plaque rupture-induced ACS is often accompanied by occlusive thrombosis.

## 3. Role of imaging in nonobstructive CAD

Approximately one-fifth of ACS cases occur despite the absence of coronary thrombi, suggesting that functional changes other than thrombus formation may contribute to its development. MI with nonobstructive coronary artery disease (MINOCA) is a condition in which MI is diagnosed based on elevated levels of cardiac enzymes and symptoms without evidence of obstructive coronary artery disease on invasive coronary angiography (ICA). The following three diagnostic criteria for MINOCA were proposed in a position paper by the European Society of Cardiology: (1) AMI criteria as defined by the Third Universal Definition of Myocardial Infarction with clinical evidence of ischemic symptoms; (2) absence of *a* > 50% stenotic lesion in the major epicardial vessels; and (3) absence of other specific causes for the acute clinical symptoms (41). Various mechanisms have been postulated for the development of MINOCA, including plaque disruption, epicardial coronary vasospasm, coronary microvascular dysfunction, coronary embolism/thrombosis, CAD, spontaneous coronary artery dissection, and supply-demand mismatch (42). MINOCA occurs in 6%–8% of patients diagnosed with acute MI. MINOCA is more common in women and often presents as non-STEMI. Imaging modalities such as CMR and intravascular imaging are reportedly useful in investigating the underlying cause of MINOCA (42). Simultaneous assessment of CFR or coronary microvascular resistance and detection of ischemia is recommended during catheter laboratory testing in patients with ischemia with nonobstructive coronary arteries (INOCA) (43). Comprehensive assessment of the structure, ischemia, and CMD will help provide better CCS management (21, 44).

**Figure 2:**
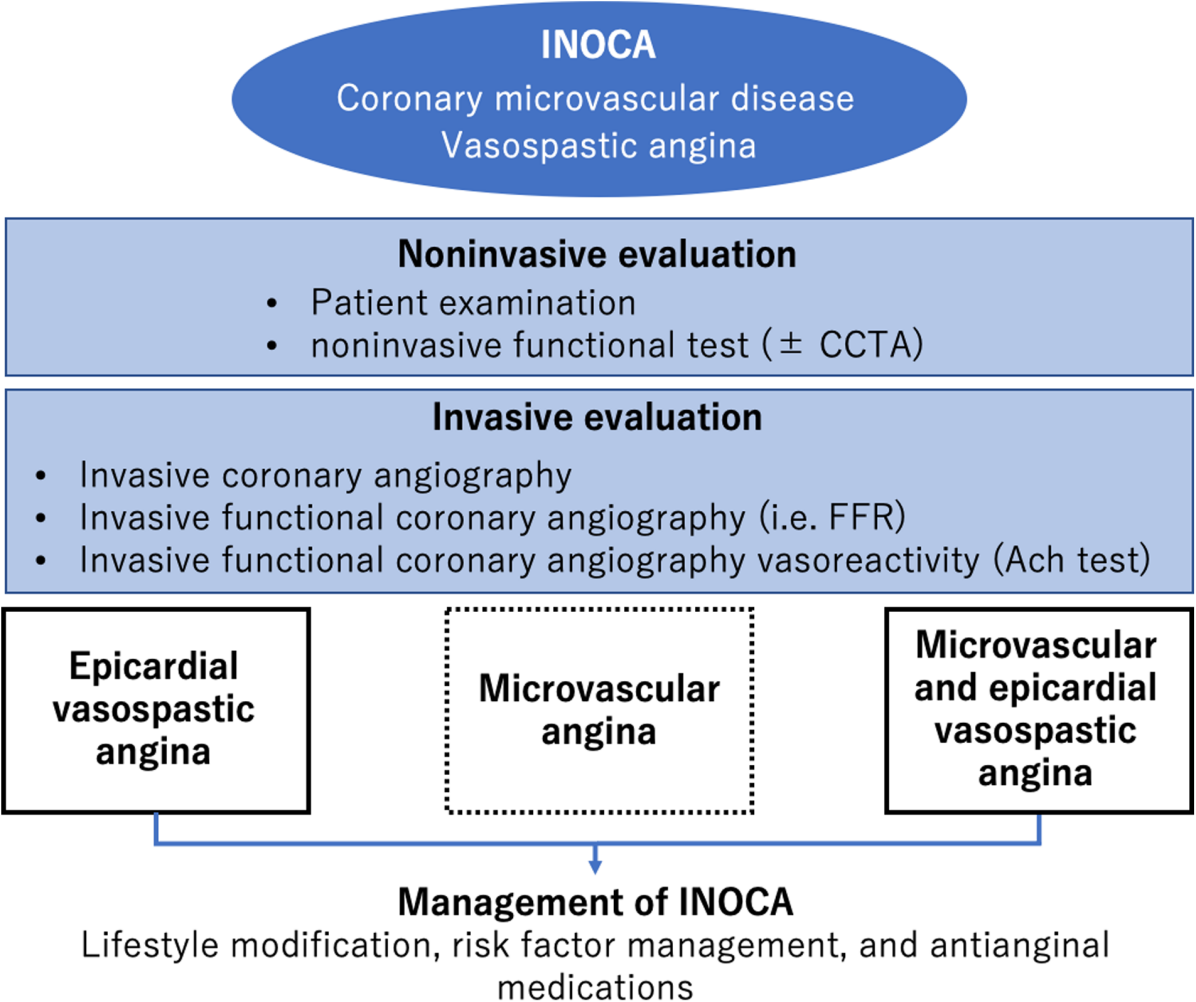
Diagnosis and management of INOCA. Modified from reference (47).

There is a growing interest in INOCA (12, 45, 46), a condition in which there is no significant stenosis in the epicardial coronary artery on ICA despite anginal symptoms and ischemic findings on noninvasive testing. Figure 2 illustrates the diagnostic strategy for INOCA (47). The pathogenesis of vasospastic angina (VSA) involves abnormal endothelial function, that is, decreased production of nitric oxide by endothelial cells, which is a scenario encountered in CCS (38). VSA treatment includes smoking cessation and administration of calcium channel blockers and vasodilators. Another condition of INOCA is CMD, which is more common in women than in men and involves organic microvascular narrowing and coronary spasm (21). The diagnostic criteria of CMD are defined by the Coronary Vasomotor Disorders International Study (COVADIS) group (44): exertional chest pain or dyspnea, absence of obstructive CAD, objective evidence of myocardial ischemia on functional imaging, and CMD as determined by measuring CFR or coronary microvascular resistance (21). Coronary microcirculatory function can be indirectly assessed using noninvasive imaging modalities, including transthoracic doppler echocardiography (48, 49), CMR (50), and positron emission tomography (PET)/computed tomography (CT) (51, 52). Moreover, a comprehensive assessment of CFR and coronary microvascular resistance, in addition to ischemia detection, is recommended in the catheter laboratory when diagnosing INOCA (43).

## 4. Functional or structural imaging

Patients with a history of MI and anginal symptoms are reportedly at high risk of cardiovascular events and may require more intensive treatments (53). For patients with a high pretest probability, evaluation of myocardial ischemia is important for patient management. For several decades, physicians have largely depended on the identification of patients with myocardial ischemia to perform coronary revascularization (54). Functional imaging for identifying myocardial ischemia includes single photon emission computed tomography (SPECT), PET/CT, stress echocardiography, or stress CMR. Although these imaging modalities are highly useful with high diagnostic accuracy for obstructive CAD in clinical practice, their ability to diagnose diffuse nonobstructive CAD is limited (55, 56).

CCTA is a noninvasive imaging technique that enables visualization of cardiac structures, coronary plaque structure and composition, and functional stenosis severity. Table 1 summarizes the recent clinical trials investigating the utility of CCTA in patients with stable chest pain. CCTA reportedly offers a high negative predictive value for significant obstructive CAD and has become a first-line test for symptomatic patients with suspected CAD (59), asymptomatic patients with low-to-intermediate cardiovascular risk (59), and those with low pretest probability (2). The strength of CCTA is that it provides direct visualization of the entire coronary artery and determines the presence of nonobstructive CAD. In the PROMISE (A Randomized Comparison of Anatomic vs. Functional Diagnostic Testing Strategies in Symptomatic Patients with Suspected Coronary Artery Disease) trial which investigated stable symptomatic outpatients referred for non-invasive evaluation of suspected CAD (*n* = 10,003), there was no significant difference between the two groups (randomized to anatomical testing with CCTA or functional testing) in the primary outcome (7). Further investigation demonstrated that CCTA had a higher discriminatory ability to predict outcomes than functional testing (57). This finding may be explained by the fact that CCTA enables the identification of nonobstructive diseases that develop into ACS and obstructive CAD requiring coronary revascularization (55, 56).

**Table 1:** Summary of the randomized controlled trials using CCTA in patients with stable chest pain.

Authors Reference	Year	Country, follow-up (years)	Study design, data source	Sample Size	Comparison	Primary endpoint	Outcome		Summary of findings
U Hoffman et al. Reference (57)	2017	North America, 26.1 months	PROMISE trial, prospective observational study	9,102	CCTA vs. functional	A composite of time-to-MACE including death from any cause, MI, or hospitalization for unstable angina	CCTA vs. functional death, 137 (3.1%) vs. 132 (3.0%). Death, 62 (1.4%) vs. 66 (1.4%)., MI, 26 (0.6%) vs. 31 (0.7%)., Unstable angina, 52 (1.2%) vs. 41 (0.9%).		No difference in clinical outcome
De Newby et al. Reference (8)	2018	Scotland, 4.8 y	SCOT-HEART trial, prospective observational study	4,138	SOC vs. SOC+CCTA	Death due to CAD or nonfatal MI	SOC vs. SOC +CCTA group 2.3% (48 patients) vs. 3.9% (81 patients)		The use of CCTA resulted in a significantly lower rate of death due to CAD or nonfatal MI.
JM Lee et al. Reference (56)	2019	Korea, 5 y	The 3 V FFR-FRIENDS study,prospective observational study	299 (772 vessels)	High risk plaque characteristics ≧3 vs. <3 with FFR	VOCO (ischemia-driven target vessel revascularization, vessel-related MI, and cardiac death)	The cumulative incidence of VOCO at 5 years was 4.3%, 15.0%, and 10.7% among the deferred vessels with FFR >0.80 and ≥3 high-risk plaque characteristics, and stented vessels with FFR ≦0.80, respectively.		Integration of both physiological stenosis severity and plaque vulnerability would provide better prognostic stratification of patients than the individual components alone.
E Sorbets et al. Reference (53)	2020	45 countries, 5 y	CLARIFY registry, the prospective observational study	32,703	Prior MI vs. no prior MI Angina vs. no angina	CV death and nonfatal MI	Prior MI	CV death or non-fatal MI 9.1% vs. 6.4% (Prior MI) PCI 7.1% vs. 7.9%	Both angina and prior MI are easily identifiable high-risk groups
							Angina	CV death or non-fatal MI 9.8% vs. 7.5% (Angina) PCI 9.6% vs. 6.8%	
MJ Budoff et al. Reference (58)	2020	USA, 18 months	EVAPORATE trial, randomized, double-blind, placebo-controlled trial	80	Icosapent ethyl (IPE) vs. placebo	The change in LAP volume measured on CCTA	IPE vs. placebo Change in LAP −0.3 mm^3^ vs 0.9 mm^3^(*P* = 0.006)		IPE significantly regressed the LAP volume on CCTA compared to the placebo.

SOC, standard of care; CAD, coronary artery disease; CV, cardiovascular; MI, myocardial infarction; ACS, acute coronary syndrome; LAP, low-attenuation plaque; CCTA, coronary computed tomography angiography; VOCO, vessel-oriented composite outcome; PCI, percutaneous coronary intervention; FFR, fraction flow reserve.

To test the hypothesis that an early invasive treatment strategy would reduce events in patients with moderate or greater ischemia rather than a conservative pharmacotherapeutic treatment strategy, the ISCHEMIA trial included cases of stable angina with documented moderate-to-severe (≥10%) ischemia (6). Blinded CCTA was performed, and patients with left main coronary artery lesions were excluded. A total of 5,179 patients from a total of 8,518 were randomized, with 2,588 patients in the invasive treatment group and 2,591 in the conservative treatment group (mean age, 64 years; 40% had diabetes and 90% had anginal symptoms). The invasive treatment group consisted of patients who underwent diagnostic catheterization within approximately one month if the core laboratory demonstrated ≥10% ischemia with exclusion of left main CAD on CCTA; PCI or CABG was performed within three months, if necessary. The conservative treatment group did not require imaging tests or invasive treatment but was continued on OMT, with a primary focus of controlling the patients’ symptoms. The follow-up period for both groups was 3.3 years, with very high follow-up rates of 99.4% and 99.7%, respectively. These results suggest that OMT may be an appropriate option for patients with CCS who meet the inclusion criteria of the ISCHEMIA trial with SPECT and CCTA. Extended analysis will provide further insights into the appropriate management of patients with CCS (60).

## 5. Non-invasive plaque imaging

**Figure 3:**
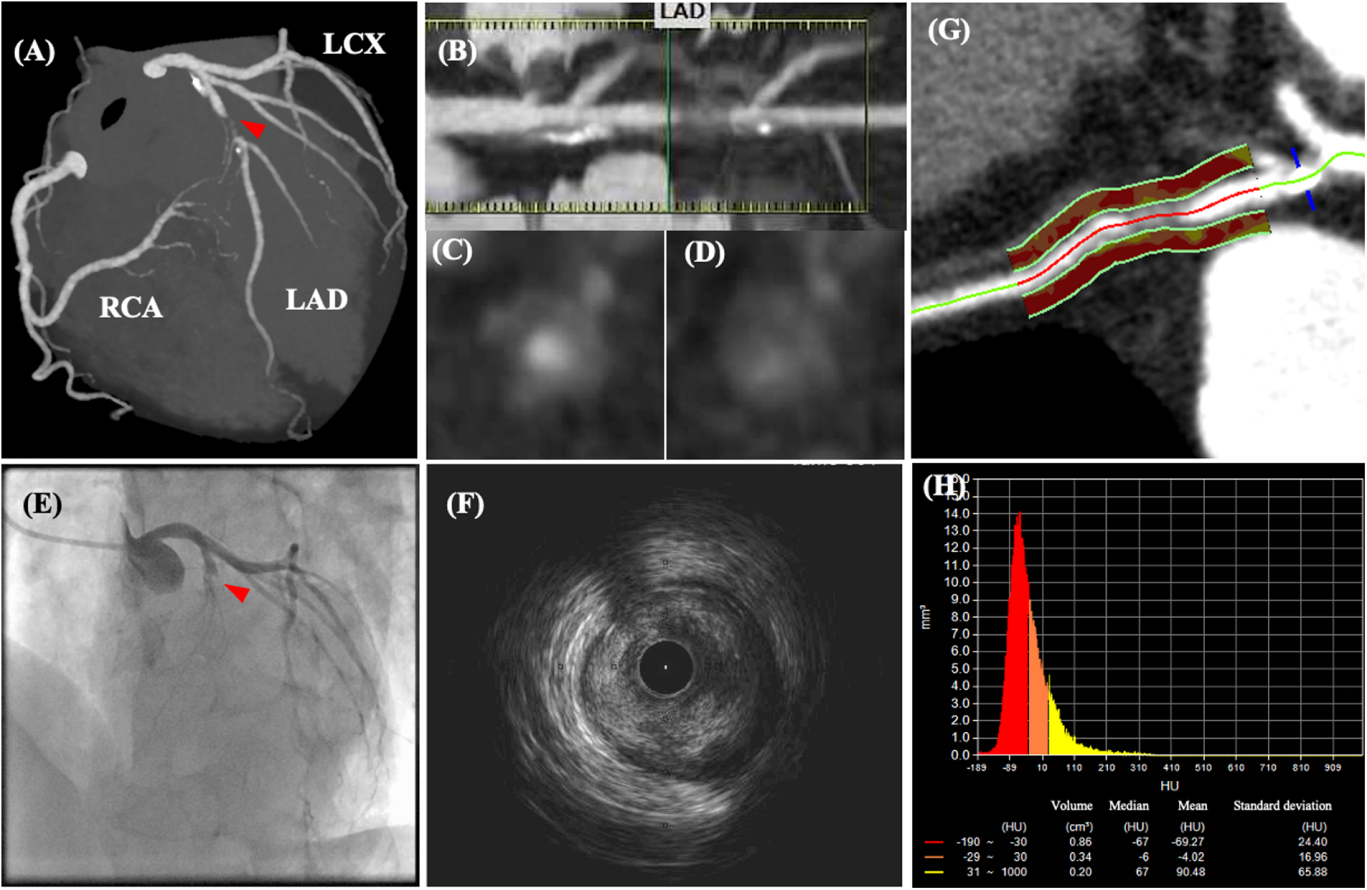
Coronary computed tomography angiography and angiographic images of a patient with acute chest pain. CCTA images of a patient with acute chest pain without ST-segment changes on electrocardiography. CCTA images (**A–D**) indicate 70–99% luminal stenosis with high-risk plaque features (positive remodeling, low-attenuation plaque, napkin ring sign, and spotty calcification) in the proximal left ascending coronary artery (LAD). After CCTA examination, the patient’s chest pain worsened, and ST-segment elevation was detected. (**E**) Emergent coronary angiography revealed total occlusion of the LAD, which corresponded to the location of the high-risk plaque visualized on CCTA. (**F**) Intravascular ultrasonography revealed an intraluminal thrombus secondary to plaque rupture. (**G,H**) Post-hoc analysis demonstrated an increased pericoronary artery attenuation of >−70 HU. Modified from reference (61).

The utility of CCTA-guided management has been well-documented. The SCOT-HEART (Scottish Computed Tomography of the Heart) trial demonstrated that CCTA-guided therapy provides better clinical outcomes than standard therapy does in patients with CCS (8). This can be explained by the effects of aspirin, statins, coronary revascularization, and lifestyle modifications through the identification of the presence of coronary atherosclerotic plaques on CCTA. Figure 3 illustrates a representative CCTA image of a patient with chest pain, showing high-risk plaque features, which led to ACS. The ROMICAT-II (Multicenter Study to Rule Out Myocardial Infarction by Cardiac Computed Tomography) trial demonstrated that high-risk coronary plaque features, including positive remodeling, low-attenuation plaques (LAP), spotty calcification, and napkin-ring sign, were independent predictors of ACS; these serve as useful diagnostic tools to rule out ACS in clinical practice (62). Although the predictive value of high-risk plaque features is not high enough to predict long-term ACS prognosis, a recent clinical trial demonstrated that a LAP volume of >4% is the strongest predictor of clinical risk factors, plaque volume, and stenosis severity (17). CCTA-derived high-risk plaques are associated with an increased incidence of ACS even in patients without myocardial ischemia (55). The combination of fraction flow reserve-CT and coronary structural features may provide a comprehensive assessment of patients requiring coronary revascularization and future ACS events (63).

CMR can assess perfusion and wall motion abnormalities of the left ventricle and cardiac structures, serving as the gold standard noninvasive imaging technique for diagnosing cardiomyopathy (64, 65), myocarditis (66), MI, and mechanical complications of MI (67). CMR with late gadolinium enhancement also allows the visualization of scar tissue and enables differentiation of recent MI from prior MI (68). CMR with T1 mapping is an alternative method for assessing myocardial edema (69, 70). CMR is widely used to differentiate other diseases mimicking ACS, including Takotsubo cardiomyopathy (65, 71) and MI with MINOCA (42). CMR also allows visualization of coronary plaques by assessing the signal intensity (72, 73). Hyperintense plaques, defined as a plaque-to-myocardium signal intensity ratio of >1.4, reportedly predicts ACS events in patients with suspected or known CAD (72). Daniel et al. demonstrated an association of intraplaque hemorrhage-related unstable carotid plaque features with stroke and myocardial infarction in 1,349 patients without a history of stroke or CAD with subclinical atherosclerosis. This indicates that the presence of intraplaque hemorrhage in the carotid arteries is associated with stroke and CAD development, independent of plaque size or cardiovascular risk factors (74, 75).

PET/CT imaging with a variety of radioactive tracer probes is used to identify and characterize arterial plaque burden at high risk for rupture and subsequent thromboembolic vessel occlusion (76). Peripheral vascular and coronary inflammation can be detected and quantified using 18-F-fluorodeoxyglucose (18F-FDG)-PET/CT (77). However, in approximately 50% of patients with ACS or MI, no local increase in coronary 18F-FDG uptake is observed. Thus, a less inflammatory but lipid-rich coronary plaque burden may account for a significant portion of the coronary plaque ruptures. Additionally, 18F-FDG-PET/CT scans may be negative in lipid-rich plaques because inflammation-induced macrophage infiltration, a major substrate for 18F-FDG uptake, is less pronounced in the surviving hypoxic cells. ^18^F-fluoromisonidazole (FMISO) changes to a more reactive form and remains intracellular by covalently binding to intracellular molecules. Thus, while using 18F-FMISO to signal hypoxia, PET in combination with 18F-sodium fluoride (18F-NaF) can determine active calcification in the CAD process and microcalcifications (78, 79).

## 6. Intracoronary plaque imaging

ICA, the gold standard for assessing CAD severity, enables visualization of the coronary arterial lumen; however, its ability to assess the outer vessel walls is limited. Intravascular ultrasound (IVUS), optical coherence tomography (OCT), and angioscopy are used to elucidate the pathogenesis of ACS and progression of coronary atherosclerosis. The use of intracoronary imaging in PCI guidance is increasing (80), thereby revealing the post-interventional mechanisms of stent failure, including thrombosis and restenosis (81, 82). Dual antiplatelet therapy (DAPT) effectively prevents post-implantation stent thrombosis; however, bleeding is the primary, major complication ofcoronary revascularization (83). Complex PCI involves intervention in patients with left main disease, multiple stent implantations, and severely calcified lesions, often requiring a longer duration of DAPT (84, 85). Clinical guidelines recommend the potential benefit of an antiplatelet-anticoagulation combination therapy or anticoagulation monotherapy (86). Furthermore, recent clinical studies have demonstrated that intracoronary imaging may beneficially affect the outcomes of patients who undergo PCI (87). Whether antiplatelet or anticoagulation therapy is effective in preventing device-oriented complications in patients undergoing complex PCI remains controversial.

IVUS assesses the coronary plaque morphology, arterial lumen, and vessel wall size, which improves our understanding of the pathogenesis of ACS (88–90). In addition to advances in the faster pullback of grayscale IVUS, virtual histology (VH-), integrated backscatter (IB-), and near-infrared spectroscopy (NIRS-) IVUSs allow the evaluation of atherosclerotic tissue characteristics. In the PROSPECT trial using VH-IVUS, 697 patients with ACS were studied; plaque volume of >70%, VH-thin-capped fibroatheroma (TCFA), and minimal luminal area of <4 mm^2^ were predictors of lesions associated with a 3-year incidence of major cardiovascular adverse events (MACE) (16). However, IVUS’s (resolution: 100–200 µm) ability to detect fibrous cap thickness in TCFA (<65 µm) is limited. NIRS–IVUS is an imaging technique based on near-infrared spectroscopy that assesses the probability of lipids being present as a chemogram (91). A multicenter prospective study investigating 1,563 patients showed that NIRS–IVUS enabled the identification of patients with a high probability of developing MACE (92). Over a mean follow-up of 732 days, the incidence of MACE in patients with maxLCBI4mm ≥400 without significant stenosis was 13%, which was twice as high as that in patients without maxLCBI4mm ≥400 (6%). These clinical studies indicate that the lipid/necrotic core burden may be a potential therapeutic target.

**Figure 4:**
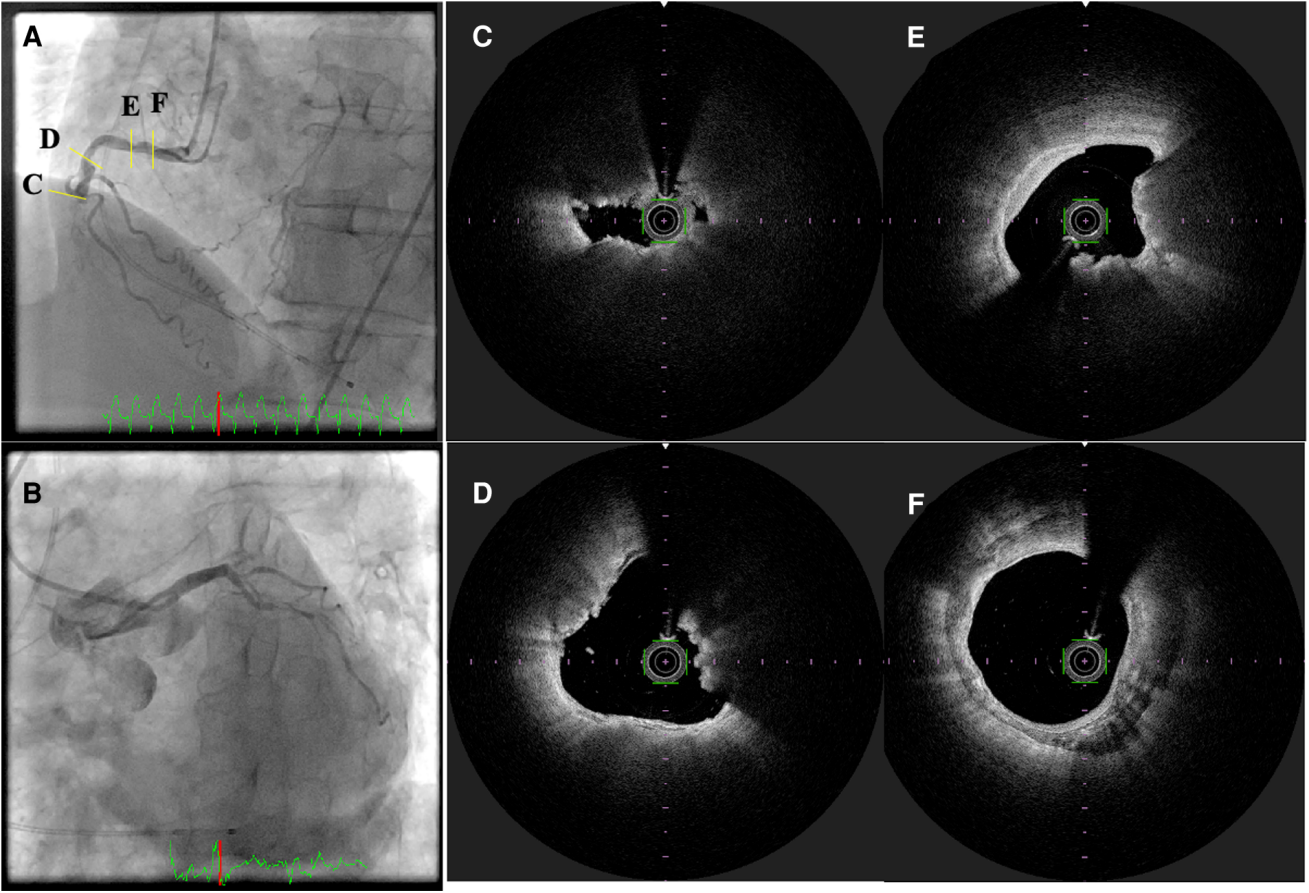
Optical frequency domain imaging of ST-segment elevation myocardial infarction. (**A,B**) Invasive coronary angiography revealed total occlusion of the mid-portion of the right coronary artery with collateral flow to the left descending coronary artery. (**C–F**) optical frequency domain imaging (OFDI) images after thrombectomy. (**C**) Thin capped fibroatheroma, (**D,E**) calcified nodules (yellow arrows), and (**F**) calcification.

OCT uses near-infrared light to image the structures of the coronary vessel walls with a high resolution of 0–15 µm (93) (Figure 4). OCT can investigate tissue response and stent expansion or apposition during PCI; however, it requires blood clearing during procedures. Recent clinical trials have demonstrated the non-inferiority of OCT compared to IVUS and its superiority over ICA alone (94). This indicates that intravascular imaging-guided PCI has advantages over angiography-guided PCI in patients with ACS (95) and CCS (96). OCT aids in visualizing the microstructures of the coronary arterial walls, including the fibrous cap (97), lipid content (98, 99), calcification (100), macrophages (101, 102), cholesterol crystals (103, 104) and neovascularization (105–107). In the CLIMA study (108), Prati et al. investigated the prognostic value of OCT findings of lesions in the left descending coronary artery. In a total of 1,776 lipid plaques, the presence of MLA <3.5 mm^2^, fibrous cap thickness <75 µm, lipid arc circumferential extension of >180°, and OCT-defined macrophages were associated with an increased risk of the primary endpoint. Despite the substantial association between the presence of TCFA and MACE, OCT-TCFAs do not necessarily lead to ACS (99). Most thrombus formation associated with plaque rupture or erosion without clinical events is thought to be remodeled by vessel healing, followed by progression of the stenosis grade (40, 109). These findings have motivated the introduction of novel intracoronary imaging modalities, such as near-infrared autofluorescence and near-infrared fluorescence OCT (110), dual-modality OCT-IVUS (111), and polarization-sensitive (PS-) OCT (112, 113) to investigate the biological tissue components and comprehensive structures of coronary atherosclerosis (114, 115).

## 7. Lipid-lowering therapy in CAD

A meta-analysis of randomized clinical trials (RCT) investigating the effects of intensive lipid-lowering therapy found that statin therapy caused regression of the atherosclerotic disease burden (116). Other RCTs have also demonstrated that for patients at a higher risk of atherosclerosis, lower LDL cholesterol levels were better for plaque regression and CAD prognosis (117–119). Ezetimibe, a commonly used non-statin lipid-lowering drug, reduces LDL cholesterol levels by 13%–20%, with a low incidence of side effects. The IMPROVE-IT (Improved Reduction of Outcomes: Vytorin Efficacy International Trial) trial demonstrated that the addition of ezetimibe to statin therapy resulted in a progressive reduction in LDL cholesterol levels and improved cardiovascular outcomes (118).

The role of imaging in the investigation of the effects of lipid-lowering therapies in patients with CAD is being increasingly recognized. In the FOURIER trials, Sabatine et al. demonstrated that PCSK9 inhibition with evolocumab, a monoclonal antibody, lowers the LDL cholesterol levels, leading to a reduced risk of cardiovascular events. The effect of alirocumab, a PCSK9 inhibitor, on the plaque volume and characteristics were evaluated in the PACMAN-AMI (Effects of the PCSK9 Antibody Alirocumab on Coronary Atherosclerosis in Patients With Acute Myocardial Infarction) randomized trial (15). The mean change in atherosclerotic volume assessed using IVUS was −2.13% in the alirocumab group and −0.92% in the placebo group. The change in the minimal fibrous capsule thickness was also more in the alirocumab group than in the placebo group, proving its plaque-reducing effect.

CCTA is an imaging modality widely used to study the pharmacological effects of changes in the plaque volume and composition. Budoff et al. demonstrated that icosapent ethyl reduced LAP volume (<50 HU) on CCTA over 18 months (58). Furthermore, in 857 patients undergoing serial CCTA imaging, van Rosendael et al. investigated the compositional changes in the untreated, progressed coronary lesions, including low-attenuation (−30 to 75 HU), fibro-fatty (76–130 HU), fibrous (131–350 HU), low-density calcium (351–700 HU), high-density calcium (701–1,000), and 1 K (1,000 HU) plaques (11). Serial CCTA imaging demonstrated that statin therapy is associated with a decrease in low-attenuation and fibrofatty plaques and a greater progression of high-density calcium and 1 K plaques. Taken together, CCTA is a useful imaging modality that assesses coronary structures and disease burden and its changes in response to OMT and lifestyle modifications.

## 8. Anti-inflammatory therapy and future perspectives of imaging

Although experimental studies have demonstrated a causal relationship between vascular inflammation and atherosclerosis, until recently, no robust evidence has suggested that anti-inflammatory therapy can prevent adverse cardiovascular outcomes (120). Table 2 summarizes the recent clinical trials that have investigated the effects of anti-inflammatory therapy in patients with CAD. In the CANTOS (Canakinumab Antiinflammatory Thrombosis Outcome Study) trial which evaluated the effects of inflammation-targeted therapy in patients with stable CAD at residual inflammatory risk, high doses of canakinumab produced a 15% reduction in MACE and a 17% reduction in cardiovascular events (121). The CIRT trial, a prospective RCT consisting of 4,786 patients with stable atherosclerosis and diabetes or metabolic syndrome, demonstrated that low-dose methotrexate did not reduce MACE (122). However, low-dose methotrexate reduces plasma IL-1β, IL-6, and CRP levels, supporting the concept that adequate inhibition of the innate immune pathway is necessary to ensure long-term cardiovascular benefits.

**Table 2:** Clinical trials targeting inflammatory pathways.

Authors References	Year	Country, follow-up (y)	Study design, data source	Sample size	Study population	Comparison	Primary endpoint	outcome	Summary of findings
PM Ridker et al. Reference (121)	2017	39 countries, 3.7 years	CANTOS, a randomized, double-blind trial	10,061	Stable CAD, persistent elevation of hsCRP (>2 mg/l)	Three doses of Canakinumab (IL-1β antibody) 50 mg, 150 mg, 300 mg vs. placebo	Nonfatal MI, nonfatal stroke, or cardiovascular death	Placebo: 4.50 events per 100 person-years. 50 mg/150 mg/300 mg dose of canakinumab: 4.11/3.86/3.90 events per 100 person-years, respectively.	Canakinumab lowered the plasma CRP, IL-1 and IL-6 levels. Reduction in CV events.
PM Ridker et al. Reference (122)	2019	North America, 2.3 years	CIRT, a randomized, double-blind trial	4,786	Stable CAD and persistent evidence of inflammation, type 2 diabetes or metabolic syndrome	Low-dose (15–20 mg) methotrexate (a purine metabolism inhibitor) once per week vs. placebo	Nonfatal MI, nonfatal stroke, or cardiovascular death, hospitalization for unstable angina	Methotrexate group/placebo group: 201/207 patients. Incidence rate: 4.13/4.31 per 100 person-years (Methotrexate/placebo)	Halted prematurely for futility. No change in plasma IL-1β, IL-6 and hsCRP levels. No reduction in CV events
JC Tardif et al. Reference (123)	2019	12 countries, 22.6 months	COLCOT, A randomized, double-blind trial	4,745	Recent MI (<30 days)	Low-dose (0.5 mg/day) colchicine (a tubulin disrupter) vs. placebo	A composite of death from CV, resuscitated cardiac arrest, MI, stroke, or urgent hospitalization.	Colchicine/placebo incidence: 5.5%/7.1%	Reduction in CV death and CV events. Increase in pneumonia
SM Nidorf et al. Reference (124)	2013	Australia, 36 months	LoDoCo, a prospective, randomized, observer-blinded trial	532	Stable CAD	Low-dose (0.5 mg/day) colchicine plus usual care or standard care alone	The composite incidence of ACS, out-of-hospital cardiac arrest, or non-cardioembolic ischemic stroke.	Colchicine /placebo: 5.3%/16%	Colchicine effectively prevents cardiovascular events in patients with stable coronary disease.
Nidorf SM et al. Reference (125)	2020	Australia, Netherlands, 28.6 months	LoDoCo2, A randomized, controlled, double-blind trial	5,522	Chronic CAD	Low-dose (0.5 mg/day) colchicine plus usual care or standard care plus placebo	A composite of CV death, spontaneous MI, ischemic stroke, or ischemia-driven coronary revascularization.	Colchicine group/placebo group 6.8%/9.6% (Incidence, 2.5 vs. 3.6 events per 100 person-years)	Reduction in CV events

ACS, acute coronary syndrome; CAD, coronary artery disease; CV, cardiovascular; MI, myocardial infarction; hsCRP, high-sensitive C-reactive protein; IL, interleukin.

COLCOT and LoDoCo2 are two large clinical trials that provided and confirmed the hypothesis that repurposed colchicine is an effective anti-inflammatory agent in atherosclerosis (123, 125). Colchicine is an antimitotic agent that inhibits tubulin polymerization and microfibrinolysis. Part of its anti-inflammatory effect is due to its inhibition of NLRP3 inflammasome formation, which indirectly suppresses IL-1β activation and decreases the downstream IL-6 and CRP levels. The COLCOT trial included approximately 5,000 patients with ACS who either received colchicine at 0.5 mg/day or placebo. During the 2-year follow-up, the colchicine group showed a 23% reduction in cardiovascular events. These clinical trials provided evidence for the addition of anti-inflammatory therapy to standard medical regimens, and suggested the importance of imaging techniques to assess vascular inflammation and plaque stabilization (10, 126, 127).

Imaging of the inflammation for the risk stratification can determine patients at a higher risk of atherosclerotic cardiovascular disease (ASCVD). Although PET/CT enables assessment of the inflammatory status of the large aorta, pericoronary arteries, and carotid arteries, its clinical application remains limited (128–131). CCTA enables the measurement of epicardial adipose tissue volume and composition (132, 133). Recent software developments have enabled pericoronary adipose tissue attenuation analysis (Figure 3D), which serves as a predictor of patient outcomes (18). In addition, degradation of collagen, a major component of fibrous caps, plays a pivotal role in coronary plaque healing (134). Microscopic polarization-sensitive (PS) OCT (112, 113) is used to assess plaque structure and tissue polarization. Catheter-based PS-OCT enables quantitative assessment of plaque characteristics, such as collagen, vascular smooth muscle cells, and macrophages, by measuring polarization properties (birefringence and depolarization) (135–137). Otsuka et al. demonstrated that fibrous caps of plaques in patients with ACS had lower birefringence than those of plaques in patients with stable CAD (138). These findings indicate fibrous cap integrity, which could be weakened by matrix metalloproteases-induced collagen degradation. Further studies are warranted to determine whether polarimetric signatures provide additional value for diagnosing plaque stability beyond that provided by the coronary plaque structural features (82, 137–139).

## 9. Conclusions

The identification of patients with myocardial ischemia can guide the management of stable CAD, which contributes to appropriate coronary revascularization. Systemic and vascular inflammation are potential imaging targets to assess plaque vulnerability in patients at a higher risk of ASCVD events. Novel imaging technologies will open up new avenues for the assessment of plaque vulnerability beyond the stage of ischemia.
